# Insights from the SwissRN Computational Reproducibility Hackathon 2025

**DOI:** 10.12688/f1000research.180013.2

**Published:** 2026-09-11

**Authors:** Tom Willems, Tobias Kühlwein, Matthias Voigt, Michael Kurschilgen, Daniel J Stekhoven

**Affiliations:** 1 Department of Psychology, University of Zurich, Zurich, 8050, Switzerland; 2 Institute of Psychology, University of Bern, Bern, 3012, Switzerland; 3 Faculty of Psychology, UniDistance Suisse, Brig, 3900, Switzerland; 4 Faculty of Mathematics and Computer Science, UniDistance Suisse, Brig, 3900, Switzerland; 5 Faculty of Business and Economics, UniDistance Suisse, Brig, 3900, Switzerland; 6 NEXUS Personalized Health, ETH Zurich, Zurich, 8092, Switzerland; 7 Swiss Institute of Bioinformatics, Lausanne, 1015, Switzerland

**Keywords:** Reproducibility, Hackathon, Open Science, Metascience, Computational Research, Software, Community Engagement

## Abstract

Computational reproducibility hackathons provide a hands-on opportunity for identifying barriers to reproducible research. The Swiss Reproducibility Network (SwissRN) hosted a Computational Reproducibility Hackathon at UniDistance Suisse in Brig, Switzerland, with participants from diverse scientific disciplines. 19 participants attempted to reproduce five published computational analyses, achieving at least partial success in four cases. Documentation quality, software environments, and data accessibility emerged as the most critical factors for successful reproduction. These findings inform ongoing efforts to develop practical best-practice guidance and training initiatives to improve computational reproducibility across disciplines.

## Introduction

Reproducibility is fundamental to science because it ensures that research results are transparent and verifiable. Computational reproducibility focuses on the ability to obtain consistent results using the original data and computational steps, essentially testing the robustness of the code and analysis. ^1^ Within the framework of open science, computational reproducibility is tightly connected to, yet distinct from, the broader concept of replicability, which addresses whether a similar result can be achieved using the same methods with new data. ^2^

That computational reproducibility is not a solved problem is well documented: an audit of articles published under a data- and code-availability policy was able to reproduce roughly a quarter of the computational claims examined, ^3^ 74% of over 9,000 archived R scripts failed to execute without error in a clean environment, ^4^ and a coordinated reproduction attempt on 14 articles carrying an Open Data badge rated only one as exactly reproducible. ^5^ These are just a few examples of a much broader body of evidence. Hence, hackathons are a great opportunity to educate about the problem and contribute to solving it.

At a reproducibility hackathon, the goal is to use the provided data and code, usually from a published article, to determine whether the analysis described by the authors can be repeated with consistent results. Besides confirming that the computations run as intended, this often involves checking compatibility across different operating systems and software versions. The process relies heavily on full access to both data and code, making open sharing essential. For software in particular, clear documentation and adherence to established standards and ontologies are equally important pillars of reproducible work.

Such hackathons offer excellent hands-on experience, especially for early-career researchers, who can develop good research habits before rigid routines form. Building on the success of the reproducibility hackathon satellite event at the Swiss Reproducibility Conference 2024, an independent hackathon was held at UniDistance Suisse in Brig, Switzerland on June 6, 2025. The event brought together 19 participants representing a vast range of scientific disciplines and eager to strengthen reproducible research practices. In this report, we present key insights that emerged during the event.

## Results

ReproHack is a community-run event format around *reproduction as a learning activity*, focused on organized hackathons. The ReproHack Hub is the free open-source web platform that supports it. Details of the reproduction efforts are available on ReproHack Hub ( https://www.reprohack.org/event/34/), including the individual reproduction reports. Five original research papers were submitted for the hackathon, with some authors attending and engaging in reproducing work other than their own. During a short welcome talk by the hosts/organisers, which also covered organizational details and a presentation of each submission, it was communicated clearly that the event focuses on learning opportunities rather than criticism of the paper content. This was emphasized and enforced by the presence of the authors, who freely put their work under scrutiny from others, answered questions, and provided support during the reproduction attempts. In addition, attempting the reproduction efforts in groups helped creating a collaborative and constructive atmosphere.

Participants came from diverse scientific backgrounds, and many were unfamiliar with the specific topics or computational methods involved. They were free to choose the submission they would try to reproduce based on their methodological or scientific interest. If participants ran into challenges they could not solve without the authors, it counted toward a failure to reproduce. Working in groups and having direct access to the authors created a productive and supportive environment that enabled mutual learning for both participants and authors. Because the hackathon was focused on learning, participants were encouraged to attempt reproduction in the way they saw fit, meaning they had total freedom in the choice of software environment for the reproduction. They were not encouraged to use any specific reproduction support tools. Choices were made based on the submission and based on personal preference. Detailed information about the individual attempts can be found on ReproHack.

No standardized evaluation criteria were used. Participants used ReproHack’s ‘Review’ tool for descriptive evaluation of the reproduction attempt. The tool provides different scales to rate reproducibility and options to describe both the tools they used and the challenges they ran into. The first rating was a categorical question with three options: no reproduction, partial reproduction, and full reproduction. Four of the five submissions were rated as partially reproduced ( Table 1). Participants encountered several common hurdles, including unclear or missing documentation, unspecified software dependencies, and difficulties accessing code or datasets. Differences in hardware setups also contributed to variations in results, complicating reproduction efforts. One difficulty was the lack of version documentation for packages within the used programs. This led to the inability to reproduce certain aspects as the functionality of packages changed over time. Further, certain individualized variables were only partially explained, which made it difficult to understand their exact usage and how to manipulate them. Throughout the event, participants tackled these issues and provided valuable feedback to the original authors. Many challenges appeared solvable by improving or adding metadata and clarifying documentation, while others, such as configuring containerized environments, required more technical expertise. At the end of the day, teams shared their findings and discussed obstacles and potential solutions, fostering a deeper understanding of how reproducibility can be improved in practice.

**Table 1.** Submissions to reproducibility hackathon. The Mean Reproducibility Score (MRS) was collected via the ReproHack platform and reflects subjective assessments by participants and should be interpreted as indicative rather than definitive.

Title	Content to reproduce	Software environments	MRS	Reviews
On reduced input-output dynamic mode decomposition ^6^	Reduced-order models of dynamical systems identified from input/state/output data	Matlab/Octave	2	1
Bore me (not): boredom impairs recognition memory but not the pupil old/new effect ^7^	Mixed-model analyses of an eye-tracking experiment on boredom and recognition memory	R	8	1
Closed-Form Power and Sample Size Calculations for Bayes Factors ^8^	Numerical results and figures of a closed-form power/sample-size methodology for Bayes factors	R, Docker	8	3
Gender Equity Navigator (GEN) ^9^	Answers from a retrieval-augmented language model queried over a corpus of gender equity reports	Python, external Google Cloud services (e.g. Gemini)	0	2
Wastewater monitoring of SARS-CoV-2 shows high correlation with COVID-19 case numbers and allowed early detection of the first confirmed B.1.1.529 infection in Switzerland ^10^	Variant abundance time series and early Omicron detection from SARS-CoV-2 wastewater sequencing data	Snakemake workflow with Bioconda dependency resolution	6	5

The mean reproducibility score (MRS) shown in Table 1 represents the average of all submitted reviews to the question: “How much of the paper did you manage to reproduce?”, rated on a scale of 0 to 10.

Participants shared their experiences as shown descriptively in Figure 1, expressing a mix of frustration and engagement throughout the reproduction efforts. Many participants reported frustration, but despite the difficulties, there was a clear interest and satisfaction in contributing to the important topic of reproducibility. This is apparent through keywords such as curiosity and teamwork. Many appreciated the group setting and the exchange of perspectives across different levels of expertise. In addition, participants described methodological aspects of what worked well and what did not. Managing software packages, bad documentation, and missing data were among those named for the latter, while availability of both code and data, the exchange between group members, and Large Language Models (LLMs) were named as aspects that worked well during the reproduction effort.

**Figure 1.**
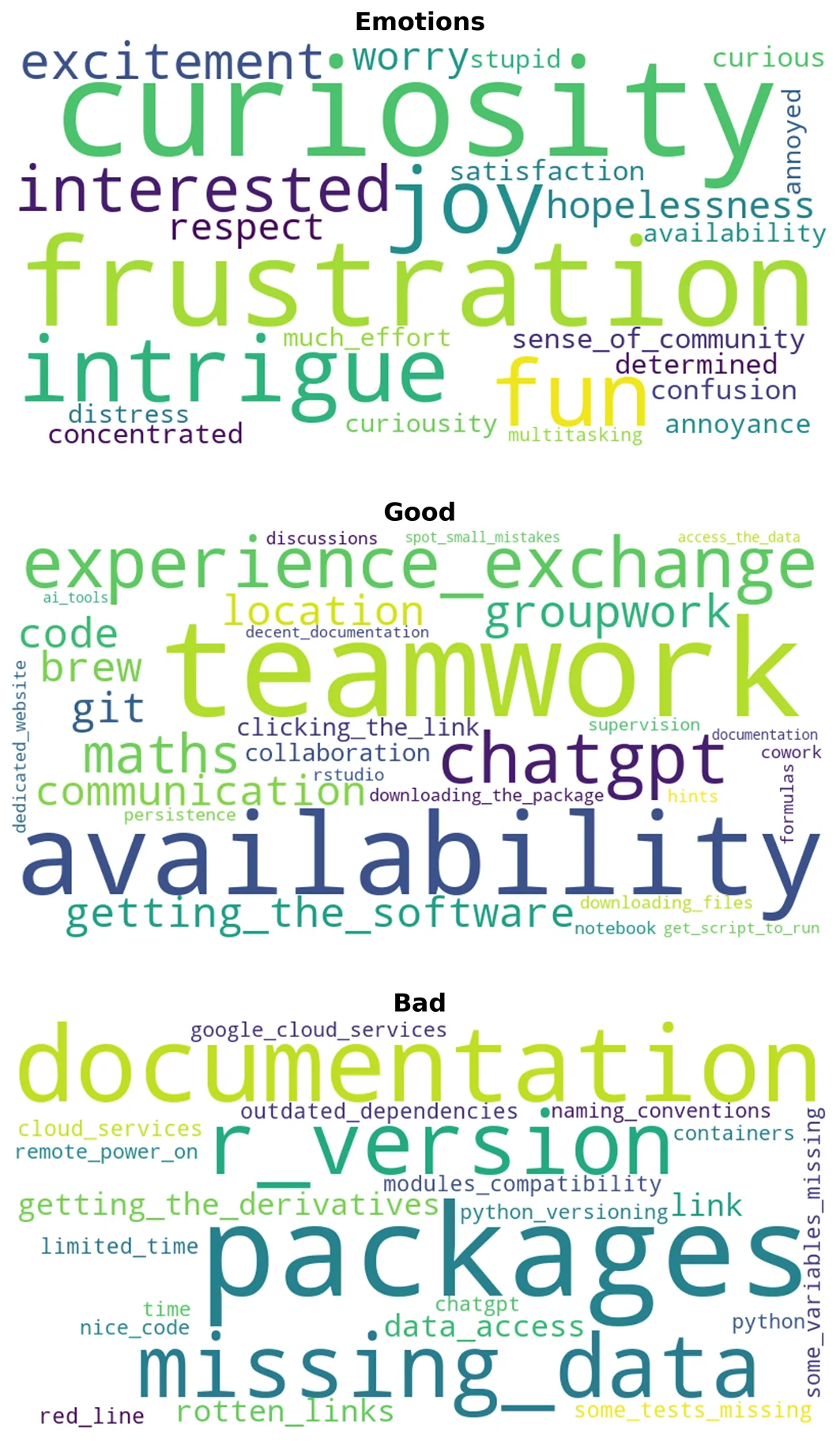
Wordles of terms submitted by participants in the final discussion of the hackathon through an online survey tool. The larger a word appears the more often it was mentioned by participants. The questions were: “What were your main emotions during the reproduction attempt?”, “What worked well?”, “What didn’t work at all?”

LLMs such as ChatGPT, were reported by participants to be helpful in creating computational environments even for those with limited technical knowledge. Outdated software and package versions proved to be significant obstacles, alongside insufficient documentation. While researchers have limited control over software stability, the responsibility for clear documentation lies with the authors. However, the lack of strong incentives often discourages investment in detailed documentation. LLMs may help ease this burden by improving clarity and readability, although their use should remain cautious and supervised: LLMs may supply plausible but fabricated rationale for analyses, so generated documentation can look authoritative while obscuring bugs. Also, it removes the comprehension check that manually writing documentation would otherwise force. Participants later emphasized, as reflected in Figure 2, that effective documentation is one of the most critical factors for successful reproducibility. By “nice code” participants referred to readability and structure, including meaningful naming, modular organisation and a transparent execution order, while “careful language” referred to precision and consistency in describing the analysis, such that variables, parameters, and processing steps are named and explained identically across manuscript, documentation, and code.

**Figure 2.**
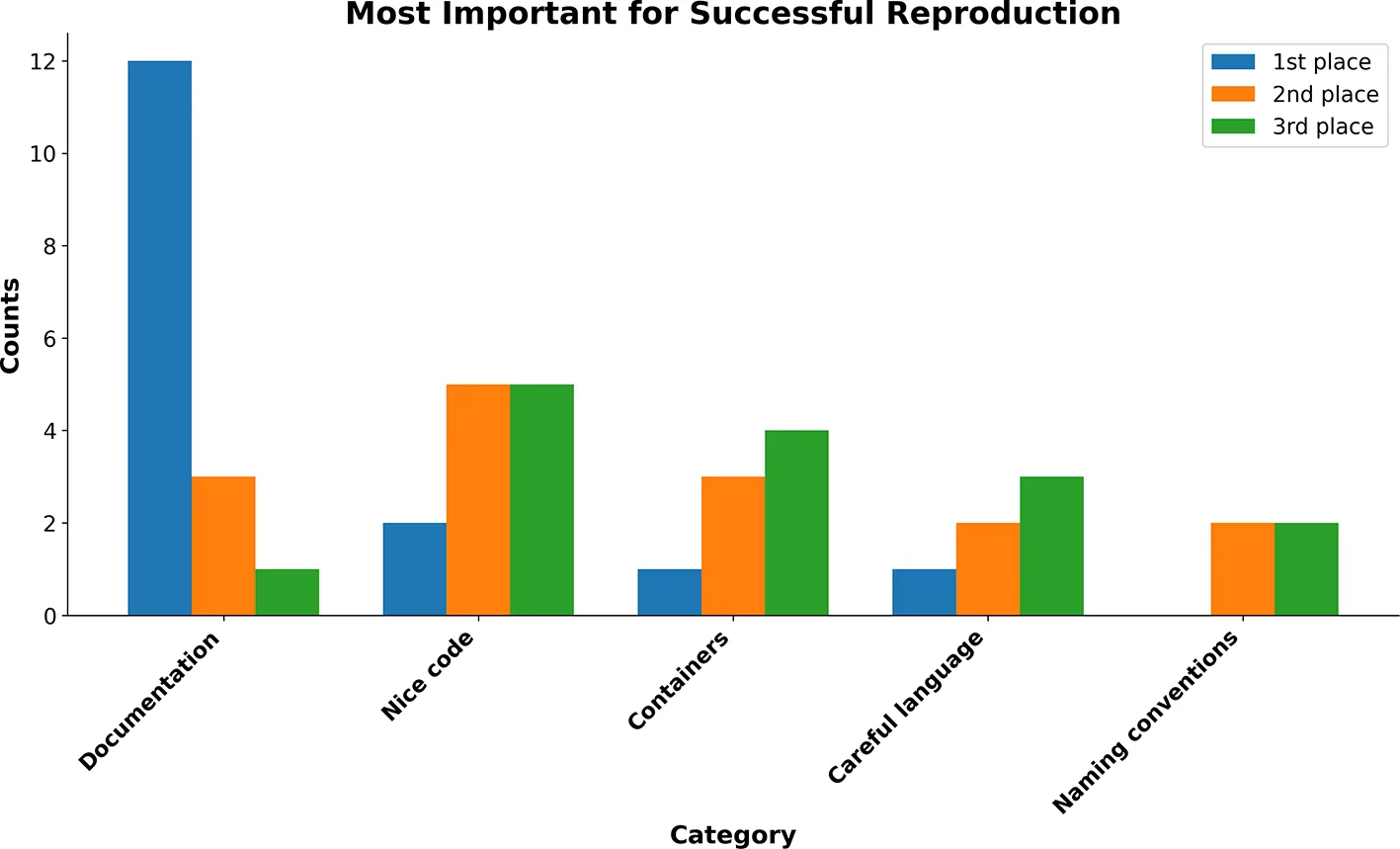
Summary of participant responses on factors enabling reproducibility.

## Discussion

The discussions at the end of the hackathon converged on a shared understanding that computational reproducibility is less a single technical hurdle and more an ecosystem problem, shaped by documentation quality, software evolution, and community norms. While participants encountered a wide range of domain-specific challenges, the obstacles they identified were remarkably consistent across projects and disciplines.

A central theme was the importance of clear, structured documentation, which participants ranked as the single most important factor when attempting to reproduce computational work. Documentation was repeatedly described as the primary entry point into a project, enabling others to understand assumptions, variable definitions, data preprocessing steps, and expected outputs. Even well-written and logically structured code proved difficult to reuse in the absence of sufficient explanatory context. This aligns with the observation that documentation often determines whether reproduction efforts can begin at all, rather than how efficiently they proceed.

Closely related to documentation, environment capture and documentation emerged as a top priority in the participant-derived checklist, receiving the highest importance rating (mean 4.4 on a 1–5 scale, tied with data accessibility and versioning). Participants emphasized that reproducibility is strongly undermined when software dependencies, package versions, or operating system assumptions are not explicitly recorded. The rapid evolution of scientific software ecosystems means that even relatively recent code may fail to execute without careful environment specification. While researchers cannot fully control external software changes, they can mitigate their impact through explicit version pinning, environment files, and containerized setups.

The same pattern has been reported at a library-hosted reproducibility hackathon, where a published analysis could ultimately be reproduced but only after participants manually resolved deprecated software libraries, and where language models proved useful in diagnosing precisely this class of error. ^11^ Together with our own experience, this suggests that dependency decay is now a routine obstacle, and that the tools available to work around it have improved.

Data accessibility and versioning were rated equally important (mean 4.4), reflecting the practical reality that reproduction efforts cannot proceed if datasets are unavailable, poorly documented, or ambiguously versioned. Participants noted that even when data were technically accessible, missing metadata, unclear preprocessing steps, or undocumented transformations created substantial barriers. Persistent identifiers for datasets and clear links between data versions and specific analyses were repeatedly highlighted as good practice.

Other checklist components (code structure, sharing, and execution (mean 3.9), result provenance and reporting (mean 3.8), and randomness control and determinism (mean 3.3)) were seen as important but secondary. This ordering suggests a pragmatic perspective: reproducibility first depends on being able to run an analysis at all, before finer-grained concerns such as numerical determinism or formal provenance tracking become relevant. Participants also ranked “nice code” and “containers” highly when asked about the most important aspects of reproduction, reinforcing the idea that readability, modularity, and standardized execution environments meaningfully lower the barrier for third-party reuse.

Our submissions spanned script-based analyses, a containerized statistical package, a cloud application, and a workflow-managed bioinformatics pipeline. Submissions with an explicitly declared, self-contained environment were reproduced most successfully, irrespective of how elaborate the underlying analysis was. The lowest outcomes arose where dependencies lay outside the reproducer's control, most clearly through reliance on credentialed cloud services and on model output that is not deterministic by design.

Importantly, our discussions underscored that reproducibility should be approached as a process designed from the outset, rather than a retrospective fix. Participants agreed that early planning – such as deciding where code and data will be hosted, how versions will be tracked, and how results will be reported – has a disproportionately large impact on downstream reproducibility. Rather than debating optimal tools, participants advocated clear, well-documented choices that can be understood and reused by others. Support structures, including research software engineers and data stewards, were identified as key enablers, particularly for researchers without formal training in software development.

Finally, the role of institutional and journal-level policies was discussed. Mandatory data and code sharing requirements were widely acknowledged as having already improved reproducibility standards, although participants noted that compliance alone does not guarantee usability, consistent with empirical work showing that shared materials are often incomplete or non-executable in practice. ^3^^,^^5^^,^^12^ In this context, lightweight tools, such as checklists, example repositories, and shared templates, were seen as promising mechanisms to translate policy requirements into meaningful practice.

## Limitations

The insights drawn from this hackathon are limited by several factors. Albeit contributing to a lively and engaging atmosphere due to a relatively small number of attendees, the small number of submissions limits the broadness of conclusions we are able to draw from this hackathon. Participants were able to ask the authors for help or discuss their submissions with them. If participants did ask the authors for help, they took this fact into account when rating the computational reproducibility. Still, the reproducibility may be skewed towards higher reproducibility due to the collaborative environment.

## Outlook

The SwissRN Computational Reproducibility Hackathon demonstrated that hands-on reproduction efforts can generate not only valuable feedback for individual projects, but also transferable insights into the structural conditions that enable or hinder computational reproducibility. Building on these experiences, a central ambition emerging from this initiative is to move beyond isolated events toward the development of widely accessible, community-driven best practices for computational reproducibility, and to embed these practices sustainably into research training.

Repeated hackathons conducted across different regions, institutions, and scientific domains, without being fixed to a specific topic, provide a particularly powerful mechanism for identifying generalizable reproducibility principles. By deliberately exposing a wide variety of computational workflows to reproduction attempts, such events allow recurring challenges and effective solutions to surface independently of discipline-specific conventions. At the same time, they naturally reveal topical or methodological special cases, which can be documented as extensions rather than exceptions to a shared core of best practices.

A key next step is therefore to distill the insights gained from multiple hackathons into a concise best-practice guide for computational reproducibility, grounded in empirical experience rather than abstract recommendations. Participant feedback from this and future events can directly inform the structure of such guidance, prioritizing aspects that have the greatest downstream impact, such as documentation quality, environment capture, and data accessibility, while offering pragmatic advice on code organization, provenance tracking, and randomness control. Making this guidance openly available in modular, reusable formats (e.g., checklists, templates, example repositories) will be essential to ensure broad adoption.

Beyond documentation, there is a clear opportunity and responsibility to translate these best practices into effective educational offerings, particularly at the undergraduate level and across disciplines. Teaching computational reproducibility early, before ad-hoc habits become entrenched, has the potential to raise baseline standards across entire research communities. Rather than treating reproducibility as an advanced or optional topic, it should be integrated into core curricula wherever computational methods are used.

To scale such efforts sustainably, we see strong alignment with established training frameworks such as The Carpentries, ^13, 14^ emphasizing reusable lesson materials, instructor training, and a “teach the teachers” model. Casting reproducibility content into this framework would allow best practices to be disseminated efficiently, adapted locally, and maintained collaboratively. In this context, close collaboration with the SwissRN Working Group for Training represents a natural and important next step, ensuring that methodological insights from hackathons are translated into pedagogically sound, high-quality training resources.

Ultimately, reproducibility hackathons should be understood as catalysts within a larger ecosystem; linking empirical assessment, community knowledge generation, and education. By systematically connecting hackathon-derived insights with open best-practice guidance and scalable training initiatives, SwissRN can contribute to a durable improvement in computational reproducibility across disciplines and career stages.

## Data Availability

Repository name: Insights from the SwissRN Computational Reproducibility Hackathon 2025. https://doi.org/10.17605/OSF.IO/3WFSQ. ^15^ The project contains the following underlying data:
• mentimeter_results.xlsx (raw, unaveraged behavioral data) • create_figs.py (script that uses behavioral data to create Figures 1 and 2 for the manuscript • figure1.tiff (Figure 1 as it is displayed in this manuscript) • figure2.tiff (Figure 2 as it is displayed in this manuscript) We do not report any extended data. Data are available under the terms of the Creative Commons Attribution 4.0 International License (CC BY 4.0, https://creativecommons.org/licenses/by/4.0/).
