# Supplementary material for: Insights from the SwissRN Computational Reproducibility Hackathon 2025
Source: F1000Res. 2026 Sep 11;15:692. Originally published 2026 May 9. [Version 2] doi: 10.12688/f1000research.180013.2 (PMC13694931; doi:10.12688/f1000research.180013.2)
Supplement: Peer review history

# Reviewer report: Reviewer response for version 2

**DOI:** 10.5256/f1000research.209785.r520282

**Published:** 2026-10-23

**Authors:** Sheeba Samuel

**Affiliations:** 1 Chemnitz University of Technology, Chemnitz, Germany

The authors have addressed the comments of the reviewer.

Is the topic of the opinion article discussed accurately in the context of the current literature?

Partly

Are arguments sufficiently supported by evidence from the published literature?

No

Are all factual statements correct and adequately supported by citations?

No

Are the conclusions drawn balanced and justified on the basis of the presented arguments?

Yes

Reviewer Expertise:

Computational Reproducibility, Knowledge Engineering, Data Provenance, Semantic Web, Data Management

I confirm that I have read this submission and believe that I have an appropriate level of expertise to confirm that it is of an acceptable scientific standard.

# Reviewer report: Reviewer response for version 2

**DOI:** 10.5256/f1000research.209785.r520281

**Published:** 2026-09-15

**Authors:** Rima-Maria Rahal

**Affiliations:** 1 Max Planck Institute for the Study of Crime, Security and Law, Germany, Germany

The revision addressed my earlier comments.

Is the topic of the opinion article discussed accurately in the context of the current literature?

Partly

Are arguments sufficiently supported by evidence from the published literature?

Partly

Are all factual statements correct and adequately supported by citations?

Partly

Are the conclusions drawn balanced and justified on the basis of the presented arguments?

Yes

Reviewer Expertise:

Decision research, Open Science

I confirm that I have read this submission and believe that I have an appropriate level of expertise to confirm that it is of an acceptable scientific standard.

# Reviewer report: Reviewer response for version 1

**DOI:** 10.5256/f1000research.198581.r490250

**Published:** 2026-06-23

**Authors:** Rima-Maria Rahal

**Affiliations:** 1 Max Planck Institute for the Study of Crime, Security and Law, Germany, Germany

In this manuscript, the authors report on the SwissRN Computational Reproducibility Hackathon 2025. I much appreciate the transparent sharing of their experiences with the community!  Nevertheless, I have some brief recommendations for improvement. 

 The introduction and discussion could benefit from a few references to pertinent literature, for those stumbling over the report without much prior knowledge about computational reproducibility. 

 I would also appreciate more details on the communication during the ReproHack. For instance, you write that “The event emphasized a constructive and collaborative atmosphere, focusing on learning opportunities rather than criticism of the paper content.”. This seems like a great idea, and other ReproHacks might benefit from your experience if they knew how exactly these guidelines were communicated. Put differently, I would appreciate a section in which you report on the details of how the ReproHack was conceived and carried out, which I think could be highly valuable for the community. 

 Moreover, Table 1 cannot be understood without reading the text - perhaps you might want to include information on what MRS is and how to interpret it in the table caption?

 For Figure 2, it would be great to know what the labels refer to specifically. For instance, what is “nice code”?  From the data shared on the OSF, I understand that the data were obtained using Mentimeter, but I could not track the questions asked or the specific answer options. Overall, for transparency and to aid understanding of how the tables and figures were obtained, it would be helpful to have the information necessary for interpretation all in one spot, perhaps akin to the classical reporting in a typical methods section.

Is the topic of the opinion article discussed accurately in the context of the current literature?

Partly

Are arguments sufficiently supported by evidence from the published literature?

Partly

Are all factual statements correct and adequately supported by citations?

Partly

Are the conclusions drawn balanced and justified on the basis of the presented arguments?

Yes

Reviewer Expertise:

Decision research, Open Science

I confirm that I have read this submission and believe that I have an appropriate level of expertise to confirm that it is of an acceptable scientific standard, however I have significant reservations, as outlined above.

# Response

**Published:** 2026-09-01

**Authors:** Tom Willems

**Affiliations:** University of Zurich Department of Psychology, Zürich, Zurich, Switzerland

**Rima-Maria Rahal**, Max Planck Institute for the Study of Crime, Security and Law, Germany, Germany

 In this manuscript, the authors report on the SwissRN Computational Reproducibility Hackathon 2025. I much appreciate the transparent sharing of their experiences with the community!  Nevertheless, I have some brief recommendations for improvement.

- *We thank Rima-Maria Rahal for the thoughtful review and the appreciation of our efforts.*

The introduction and discussion could benefit from a few references to pertinent literature, for those stumbling over the report without much prior knowledge about computational reproducibility.

- *Thank you for this helpful suggestion. We agree that readers without prior exposure to computational reproducibility need entry points into the literature. We have therefore added references for the definitional distinction between reproducibility and replicability in the opening paragraph, and the empirical evidence that computational reproduction frequently fails, which motivates events such as ours.*

I would also appreciate more details on the communication during the ReproHack. For instance, you write that “The event emphasized a constructive and collaborative atmosphere, focusing on learning opportunities rather than criticism of the paper content.”. This seems like a great idea, and other ReproHacks might benefit from your experience if they knew how exactly these guidelines were communicated. Put differently, I would appreciate a section in which you report on the details of how the ReproHack was conceived and carried out, which I think could be highly valuable for the community.

- *Thank you for the appreciation of how the hackathon was framed. We now provide some context about how these guidelines were communicated*

Moreover, Table 1 cannot be understood without reading the text - perhaps you might want to include information on what MRS is and how to interpret it in the table caption?

- *Table 1 now introduces MRS and gives context how it was collected*

For Figure 2, it would be great to know what the labels refer to specifically. For instance, what is “nice code”?  From the data shared on the OSF, I understand that the data were obtained using Mentimeter, but I could not track the questions asked or the specific answer options. Overall, for transparency and to aid understanding of how the tables and figures were obtained, it would be helpful to have the information necessary for interpretation all in one spot, perhaps akin to the classical reporting in a typical methods section.

- *Thank you for this comment. We now provide more context on the question and the individual responses. *

# Reviewer report: Reviewer response for version 1

**DOI:** 10.5256/f1000research.198581.r483839

**Published:** 2026-05-26

**Authors:** Sheeba Samuel

**Affiliations:** 1 Chemnitz University of Technology, Chemnitz, Germany

This opinion article presents reflections and lessons learned from the SwissRN Computational Reproducibility Hackathon 2025, where participants attempted to reproduce analyses from five published computational studies. The manuscript highlights practical barriers to computational reproducibility, including insufficient documentation, missing environment specifications, and data accessibility challenges. The paper also discusses the educational and community-building value of reproducibility hackathons and proposes future directions for training and best-practice development.

 Strengths:

 * The manuscript is clearly written and accessible to a broad interdisciplinary audience. 

 * The practical insights derived from hands-on reproduction attempts are valuable and potentially useful for researchers, institutions, and reproducibility initiatives.

 Weakness:

 * The manuscript does not provide sufficient methodological information about how reproduction attempts were evaluated. For example: How were participants assigned to projects?

 Were standardized evaluation criteria used?, How was the Mean Reproducibility Score (MRS) aggregated and interpreted?, What constituted “partial reproduction”?

 * Only five papers were included, limiting the strength of broader conclusions. While this is acknowledged implicitly, the limitations should be discussed more explicitly.

 * The article is primarily descriptive. Although appropriate for an opinion piece, the discussion could be strengthened by deeper synthesis or comparison with existing reproducibility literature and prior ReproHack initiatives and see if things have changed.

 * Some original authors attended the hackathon and supported reproduction attempts. While this likely improved the learning experience, it may also have influenced reproducibility outcomes. This potential bias should be acknowledged more explicitly.

 * The discussion of large language models is interesting but underdeveloped. The authors mention benefits and caution but do not elaborate on specific use cases, risks, or limitations. The authors should clarify: what kinds of reproducibility tasks LLMs assisted with, where they were beneficial, and what risks or inaccuracies may arise from relying on them.

 * The manuscript provides very limited information about the five reproduced studies beyond their titles and reproducibility scores. Readers cannot assess whether the reported challenges are generalizable because important characteristics of the reproduced papers are missing, including: computational complexity, disciplinary background, scale of the datasets, type of computational workflow, and expected technical difficulty of reproduction.For example, reproducing a statistical analysis in R differs substantially from reproducing a machine learning pipeline or a high-performance computational workflow. Without this context, interpretation of the outcomes remains difficult.

 * The manuscript emphasizes environment capture and containerization as important factors but does not report whether the submitted papers actually included: Docker/Singularity containers, Conda environments, reproducibility manifests, package lock files, CI/CD pipelines, or workflow managers.

 * The article does not specify: which programming languages were used, whether analyses relied on Jupyter notebooks, RMarkdown, standalone scripts, pipelines, or GUIs,

 or whether proprietary software was involved. These details are highly relevant because reproducibility barriers often differ substantially across computational ecosystems.

 * The manuscript mentions that participants came from diverse disciplines, but no information is provided regarding: computational expertise, programming proficiency,

 prior reproducibility experience, or familiarity with the reproduced methods. Since reproducibility outcomes are strongly influenced by user expertise, this information is necessary for interpreting the findings.

 * The manuscript does not explain how papers were assigned to participants or groups. It is unclear whether: participants self-selected projects, assignments were randomized,

 expertise matching was considered, or group sizes differed across projects.

 * The paper does not discuss whether participants used existing reproducibility-support tools.

 * Consider discussing whether barriers differed between notebook-based workflows, script-based analyses, statistical pipelines, or more complex computational infrastructures.

 * Figures 1 and 2 are referenced briefly, but the manuscript provides limited interpretation of the findings shown in these visualizations. How is 'nice code' defined? What does 'careful language' mean in this context?

 * A brief description of the ReproHack Hub platform may help readers unfamiliar with it.

Is the topic of the opinion article discussed accurately in the context of the current literature?

Partly

Are arguments sufficiently supported by evidence from the published literature?

No

Are all factual statements correct and adequately supported by citations?

No

Are the conclusions drawn balanced and justified on the basis of the presented arguments?

Yes

Reviewer Expertise:

Computational Reproducibility, Knowledge Engineering, Data Provenance, Semantic Web, Data Management

I confirm that I have read this submission and believe that I have an appropriate level of expertise to confirm that it is of an acceptable scientific standard, however I have significant reservations, as outlined above.

# Response

**Published:** 2026-09-01

**Authors:** Tom Willems

**Affiliations:** University of Zurich Department of Psychology, Zürich, Zurich, Switzerland

**Sheeba Samuel**, Chemnitz University of Technology, Chemnitz, Germany

 This opinion article presents reflections and lessons learned from the SwissRN Computational Reproducibility Hackathon 2025, where participants attempted to reproduce analyses from five published computational studies. The manuscript highlights practical barriers to computational reproducibility, including insufficient documentation, missing environment specifications, and data accessibility challenges. The paper also discusses the educational and community-building value of reproducibility hackathons and proposes future directions for training and best-practice development.

 Strengths:

 * The manuscript is clearly written and accessible to a broad interdisciplinary audience.

 * The practical insights derived from hands-on reproduction attempts are valuable and potentially useful for researchers, institutions, and reproducibility initiatives.

- *We thank Sheeba Samuel for the summary and the thoughtful review of our manuscript as well as for highlighting its strengths. *

Weakness:

 * The manuscript does not provide sufficient methodological information about how reproduction attempts were evaluated. For example: How were participants assigned to projects?

 Were standardized evaluation criteria used?, How was the Mean Reproducibility Score (MRS) aggregated and interpreted?, What constituted “partial reproduction”?

- *Thank you for this comment, we are now providing the methodological information about the reproduction attempts in more detail*

* Only five papers were included, limiting the strength of broader conclusions. While this is acknowledged implicitly, the limitations should be discussed more explicitly.

- W *e are now discussing this limitation in a new limitations chapter*

* The article is primarily descriptive. Although appropriate for an opinion piece, the discussion could be strengthened by deeper synthesis or comparison with existing reproducibility literature and prior ReproHack initiatives and see if things have changed.

- *Thank you for this suggestion. We have added references and now place our observations within the existing literature. The Introduction cites the distinction between reproducibility and replicability and the empirical evidence that computational reproduction frequently fails, In the Discussion we compare our experience with another reproducibility hackathon, where dependency decay and the usefulness of language models in resolving it were reported as well, and we now support the observation that compliance with sharing mandates does not guarantee usability with the corresponding empirical work.*

* Some original authors attended the hackathon and supported reproduction attempts. While this likely improved the learning experience, it may also have influenced reproducibility outcomes. This potential bias should be acknowledged more explicitly.

- *In a new limitations section, we are now acknowledging this fact more explicitly*

* The discussion of large language models is interesting but underdeveloped. The authors mention benefits and caution but do not elaborate on specific use cases, risks, or limitations. The authors should clarify: what kinds of reproducibility tasks LLMs assisted with, where they were beneficial, and what risks or inaccuracies may arise from relying on them.

- *We agree that the discussion of LLMs in the context of computational reproducibility is interesting and that it deserves a thorough discussion that is not attached to one single reproduction effort. The manuscript already mentions two specific use cases: helping participants to create computational environment and creating documentation, which we identified as important for reproduction success. We added potentials risks for the latter.*

* The manuscript provides very limited information about the five reproduced studies beyond their titles and reproducibility scores. Readers cannot assess whether the reported challenges are generalizable because important characteristics of the reproduced papers are missing, including: computational complexity, disciplinary background, scale of the datasets, type of computational workflow, and expected technical difficulty of reproduction.For example, reproducing a statistical analysis in R differs substantially from reproducing a machine learning pipeline or a high-performance computational workflow. Without this context, interpretation of the outcomes remains difficult.

- *We agree that interpretation of reproduction results are difficult without background info on the reproduced. We now added a column to the overview table to provide some information on the code that was subject of reproduction.*

* The manuscript emphasizes environment capture and containerization as important factors but does not report whether the submitted papers actually included: Docker/Singularity containers, Conda environments, reproducibility manifests, package lock files, CI/CD pipelines, or workflow managers.

- *This information is now also part of the overview table.*

* The article does not specify: which programming languages were used, whether analyses relied on Jupyter notebooks, RMarkdown, standalone scripts, pipelines, or GUIs,

 or whether proprietary software was involved. These details are highly relevant because reproducibility barriers often differ substantially across computational ecosystems.

- *We agree that the information about the software environments etc. is of great importance both for the reproduction attempt as well as to judge its success. We now added a paragraph that explains how decisions were made and also points the reader towards the reprohack platform where this information is noted in detail for each individual attempt.*
*  *

* The manuscript mentions that participants came from diverse disciplines, but no information is provided regarding: computational expertise, programming proficiency,

 prior reproducibility experience, or familiarity with the reproduced methods. Since reproducibility outcomes are strongly influenced by user expertise, this information is necessary for interpreting the findings.

- *As the hackathon was built around collaborative group work rather than individual reproduction attempts, participants worked in mixed teams throughout, and individual skill levels were therefore not collected and arguably less important to interpret the outcomes than for individual reproduction attempts*

* The manuscript does not explain how papers were assigned to participants or groups. It is unclear whether: participants self-selected projects, assignments were randomized,

 expertise matching was considered, or group sizes differed across projects.

- *We now explain that groups were formed based on individual preference. *

* The paper does not discuss whether participants used existing reproducibility-support tools.

- *No reproducibility-support tools were prescribed or encouraged, so choices reflect personal preference. The individual ReproHack reviews record the tools used in each attempt, and we now point the reader there.*

* Consider discussing whether barriers differed between notebook-based workflows, script-based analyses, statistical pipelines, or more complex computational infrastructures.

- *We have added a short paragraph to the Discussion comparing our submissions.*

* Figures 1 and 2 are referenced briefly, but the manuscript provides limited interpretation of the findings shown in these visualizations. How is 'nice code' defined? What does 'careful language' mean in this context?

- *We thank the reviewer for this point. We have expanded the main text accompanying Figures 1 and 2, so that the terms rated by participants are explained rather than reported verbatim, making the content of both figures more interpretable.*

* A brief description of the ReproHack Hub platform may help readers unfamiliar with it.

- *Thank you, we added a description of the Reprohack Hub platform.*
